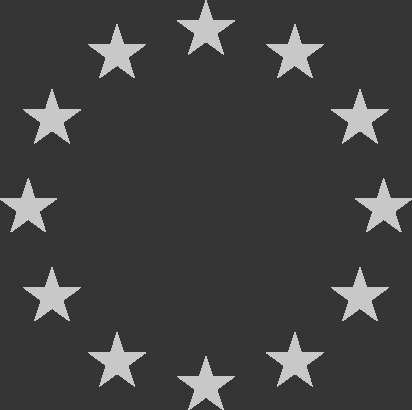# No Man’s Land? Gendering Contraception in Family Planning Advice Literature in State-Socialist Poland (1950s–1980s)

**DOI:** 10.1093/shm/hkz007

**Published:** 2019-05-06

**Authors:** Agata Ignaciuk

**Keywords:** state-socialist Poland, popular medical literature, history of contraception, gender history, gynaecology

## Abstract

This article examines popular medical discourses on contraception produced in state-socialist Poland following the legalisation of abortion in 1956, a time when the party state declared family planning to be a public health project. By analysing popular medical literature, I argue that the popularisation of family planning constructed and relied on gender norms that could ease anxieties about the mainstreaming of ideas relating to sexuality and contraception, as well as about gender equality in a state-socialist context. I show that the femininity constructed in Polish birth control advice was based in fertility and the physical attractiveness required to maintain a husband’s sexual interest. Although masculinity was represented as distant, egoistic and violent, experts broadcast mixed messages about the effectiveness and usefulness of popular male contraceptive methods, some of which were at times utterly demonised.

Following the decriminalisation of abortion for socio-economic reasons in Poland (1956/1959), the socialist state established the promotion of contraceptive use as a population and public health priority. Advice literature on family planning, written by doctors for lay readers, was one of the tools for this promotion. By advancing specific concepts of the masculine and feminine, books and pamphlets on contraception reflected and addressed anxieties relating to a new social and medical order.^1^ In the Polish context, this order was shaped not only by the aforementioned abortion law but also by the state policy of equality between the sexes and women’s participation in paid employment.

This article argues that Polish state-socialist prescriptive literature on contraception framed family planning as a strategical tool for achieving the health and well-being of women, families and, by extension, the state. Borrowing ideas about family planning from both sides of the Iron Curtain, Polish popular medical narratives about this strategical tool were constructed through contradictory notions of gender roles in the prevention and spacing of births. Despite their inherent contradictions, these notions remained relatively stable between the late 1950s and late 1980s. The femininity constructed within birth control literature in state-socialist Poland was linked to the fertility of a woman, to her health and that of her children, and to her physical attractiveness, a condition required to maintain a husband’s interest. Masculinity, on the other hand, was represented as distant, egoistic and violent. Experts broadcast mixed messages about the effectiveness and usefulness of popular male contraceptive methods, a number of which were essentially demonised.

This article builds upon and expands the emerging scholarship on the social and cultural history of reproductive health, medicine and activism in state-socialist Poland. Most of this literature has focused on analysis of the background, introduction and consequences of the 1956 abortion law.^2^ One of the most intensely studied aspects has been the foundation of Towarzystwo Świadomego Macierzyństwa (Society for Conscious Motherhood, henceforth SCM), the state-sponsored family planning organisation and its role in the shifting biopolitics of the party state^3^ and other local and international entanglements.^4^ This article builds and expands on work by a number of scholars, particularly the historians, Sylwia Kuźma-Markowska, Natalia Jarska and Barbara Klich-Kluczewska, and the anthropologist Agnieszka Kościańska. Kuźma-Markowska, whose work has been crucial in mapping SCM’s role and relations during the late 1950s and the 1960s, has used popular advice literature on family planning to explore the attitudes of doctors and activists to sterilisation in Poland during the interwar period and the 1950s.^5^ Jarska has reflected on Polish marital manuals as sources for examining the expertise on family and marriage as social institutions.^6^ Klich-Kluczewska, in her pioneering study of taboos bordering the institution of family—a crucial site of intervention for both the Polish party state and the Catholic Church—has studied the processes of expertisation in Poland through the lens of sociology.^7^ Kościańska has examined the delivery of expert advice during the 1970s, focusing on the consolidation of sexology as a field of scientific enquiry and social intervention.^8^ These contributions, have, however, left the changing policies and discourses on family planning and contraception largely unexplored. This is due to the fact that for sexologists and sociologists, contraception was a side rather than central issue. Placing it centre stage, therefore, sheds new light on the gender order constructed in post-war Poland, both discursively and bodily, by medicine and particularly gynaecology, which had become the key agent in the intense process of medicalising reproduction.^9^

Placing the popularisation of family planning at the centre of my enquiry also adds new perspectives to the dynamically expanding historiography of birth control movements, policies and propaganda in areas beyond the well-studied global North and South,^10^ Central and Eastern Europe included.^11^ This article also advances scholarship on the history of gender in the region, which has explored the often conflicting state-socialist proposed gender norms in contexts where official equality between men and women, and the prominent presence of the latter in the paid workforce (including medicine),^12^ was linked with the communist party’s policies emphasising women’s responsibility for children and the household, especially from the second half of the 1950s onwards.^13^ In the Polish context, these tensions between nominal equality and women’s twofold burden were further complicated by the strong presence of the Catholic Church, which actively opposed the 1956 abortion law and ensuing contraceptive propaganda throughout the state-socialist period.^14^

In what follows, I first contextualise the history of family planning popularisation in state-socialist Poland in relation to the shifting priorities of population policy. After a brief discussion of the sources used, I analyse how Polish family planning advice literature from the late 1950s to the mid-1980s constructed contraception as a legitimate practice and in what circumstances family limitation was promoted or discouraged. I then analyse ways in which this literature defined norms of femininity and examine portrayals of the division of responsibilities in the realm of family planning. Finally, I look at how the ideal masculinity represented in this literature clashed with narratives of male violence and marital rape, against which women were encouraged to use contraception as a form of self-defence.

## Abortion Law Reform and the Popularisation of Family Planning 

In this section, I establish how the popularisation of contraception became a public health project in state-socialist Poland from the late 1950s onwards. I also discuss how the SCM, a privileged agent in this project during its early years, lost its monopoly during the 1970s and how the Catholic understanding of family planning became increasingly mainstreamed as state and Church ideas converged on the need to stimulate population growth.

The ‘conditions for the legal termination of pregnancy’ established on 27 April 1956 regulated access to abortion during most of the state-socialist period.^15^ Abortion was authorised in three situations: when termination was deemed medically necessary; when there were grounds to believe pregnancy had resulted from a criminal act and when a woman found herself in ‘difficult life circumstances’. Sylwia Kuźma-Markowska argues that communist authorities and their advocates in the medical profession presented the new law as a public health measure to protect women’s lives and reproductive health, both under threat from illegal abortionists.^16^ As interwar abortion regulation had authorised termination on medical and criminal grounds, it was the socio-economic premise that considerably increased the number of legal abortions in Poland.^17^ Numbers increased further after an executive order by the Ministry of Health and Welfare in 1959 simplified the procedure, abolishing the initial requirement that hardship must be proven: a woman’s written statement now sufficed.^18^ The new regulation also obliged doctors performing terminations to instruct the woman ‘about ways of preventing an unwanted pregnancy, handing her a prescription for a suitable contraceptive method … as well as appropriate literature on contraception’.^19^ Contraceptive advice and provision were to be mainstreamed through a state-sponsored public health project in which the SCM was assigned a key role.

The name chosen for this interdisciplinary association, which brought together doctors, journalists and activists, was somewhat anachronistic in the 1950s, when ‘family planning’ was transnationally becoming the dominant framework of contraception popularisation.^20^ Yet, appealing to ‘conscious motherhood’ was a carefully planned strategy of tracing the Polish Society’s genealogy back to the interwar ‘conscious motherhood’ movement. Interdisciplinary birth control activism had blossomed in many Polish cities during the 1930s, with birth control information being delivered to the poorest classes, especially the urban proletariat, by socialist doctors, some of whom continued their activism after the Second World War.^21^ In 1931, under the rallying cry of ‘Here we prevent pregnancies, not terminate them’, socialist physician Justyna Budzińska-Tylicka (1867–1936) founded a Conscious Motherhood Clinic in Warsaw;^22^ other activists would establish a number similar clinics in cities across the country during the 1930s.^23^ For Budzińska-Tylicka—one of the first Polish women to graduate in medicine^24^—‘conscious motherhood’ meant responsible regulation of family size through ‘secure and safe contraceptives—not through artifical termination, which ruins the woman’s health’.^25^ For interwar family planners, secure and safe contraceptives denoted physician-fitted pessaries.^26^

The core goal of the state-socialist SCM, formulated during the first years of its activity (1957–59), was similar to that defined by Budzińska-Tylicka in the 1930s: to prevent abortion from being used as a birth control method. From the late 1950s onwards, this was to be attained through the production and dissemination of contraceptives, along with counselling to raise people’s awareness of family planning and their ‘sexual culture’.^27^

The SCM had close links with the British Family Planning Association and in 1958 became the first national family planning association from Central and Eastern Europe to join the International Planned Parenthood Federation (henceforth IPPF).^28^ These links, in addition to the interwar ‘conscious motherhood’ tradition, were important ingredients in the construction of SCM’s identity and crucial for providing access to the expertise necessary for domestic contraceptive production.^29^ In 1958, SCM founded a sister company, named Securitas,^30^ which would be the sole domestic provider of diaphragms, cervical caps and spermicides throughout the state-socialist period.^31^

Assigning the dissemination of contraceptive products and advice to an association linked to, but officially independent from, health authorities—rather than the Ministry of Health and Welfare or Ministry of Chemical Industry—was justified by a supposed lack of training among doctors in the *Poradnie K* network of public gynaecological clinics leaving them ill-equipped to take on the mass popularisation of contraception.^32^ However, this could be interpreted as a deliberate strategy of ‘othering’, ensuring that this highly controversial endeavour, for which the party state almost certainly anticipated opposition from the Catholic hierarchy and laity, would not be directly linked to state institutions.^33^

The Society’s domination of birth control campaigning began to decrease during the 1970s. Periodic rapprochements that occurred between the communist regime and the Catholic Church intensified during this decade, damaging public support and funding for the organisation, now renamed the Society for Family Planning (Towarzystwo Planowania Rodziny, henceforth SFP) and expected to become a part of the state-socialist ‘family counselling system’.^34^ These developments were in part motivated by both Church and state becoming convinced that population growth, which had slowed down as Poland entered the last phase of its demographic transition during the 1960s, required revitalisation.^35^ Not only had two-child families become the norm, the number of people opting for just one child was rapidly increasing.^36^ While some demographers interpreted the declining birth rate primarily in terms of urbanisation and women’s participation in paid employment, others blamed it on unrestricted contraceptive propaganda and abortion. These debates prompted a shift in population policy towards moderate pronatalism,^37^ resulting in reductions in the import and domestic production of contraceptives, and weakening of the position of the SFP, known from 1979 as the Society for Family Development (Towarzystwo Rozwoju Rodziny, henceforth SFD).

While the SCM/SFP/SFD reached out to the public through a variety of channels, including the general press, women’s magazines, radio programmes and documentary films, perhaps the most important media were large print books and pamphlets. Production of these was most intense during the early years of the Society’s activity, when it enjoyed unconditional support from the state. By 1970, the SCM had sponsored the production of over 9 million copies of publications about contraception, abortion and ‘sexual culture’.^38^ The above-mentioned shift in population policy during the 1970s, however, had a dramatic impact on the Society’s publishing activites. The withdrawal of supplies of printing paper in 1976 marked the loss of its privileged position as the executor of family planning policy.^39^ From the mid-1970s onwards, competing visions of family planning were mainstreamed, including ‘natural regulation of conceptions’, intensely endorsed by anti-abortion and anti-‘artificial’ contraception Catholic doctors such as Włodzimierz Fijałkowski, and promoted through public channels, including Państwowy Zakład Wydawnictw Lekarskich (henceforth PZWL), the state medical publisher, who had printed the majority of SCM/SFP material.

The books and pamphlets promoting contraception, family planning and ‘responsible parenthood’ that I have analysed for this article belong to the genre of popular medical literature, a traditional source for historians of medicine and health that has enriched social and cultural histories of contraception^40^ and sexuality.^41^ While, as Peter Laipson has pointed out, prescriptive literature does not necessarily reflect actual behaviour,^42^ it does depict desired conduct, as well as actions deemed to be modifiable. Thus, the subgenre of contraception-popularising literature is examined here to study how contraception was gendered and medicalised and how this gendering and medicalisation reflected emerging and shifting ideologies relating to birth control in state-socialist Poland and, more generally, in Central and Eastern Europe. In so doing, this article adds to the growing scholarship that has used marital manuals to examine the changing norms and cultures of sexuality and marriage in the region in the 19th and 20th centuries.^43^

This article is the first systematic study of family planning advice literature published in state-socialist Poland. By searching the catalogue of the National Library of Poland^44^ and studying SCM/SFP/SRR reports listing publications^45^, I identified 32 books, booklets and pamphlets aimed at the general public^46^ that either focused entirely on contraception or in which contraception was a prominent theme.^47^ In order to access state-approved discourses on contraception, formulated by experts who were at the same time executors of state policies on abortion and contraception, I prioritised those written by practising gynaecologists^48^ and printed by the state-owned medical publisher, PZWL.^49^ As discussed earlier, the lens of the SCM/SFP/SRR was particularly decisive between the late 1950s and mid-1970s, before a shift in population policy ended the Society’s domination of family planning information.

In approaching this material, I have paid particular attention to the ways in which arguments were formulated to justify the need for contraception and the gendered division of both responsibilities and recommended methods. As well as exploring the gendered tensions, contradictions and hierarchies revealed in this material, my broader aim is to analyse the continuities and ruptures relating to these arguments, responsibilities and methods over the last four decades of Polish state socialism.

To contextualise and support my analysis of contraception advice literature, I also explored other channels through which contraception was popularised in state-socialist Poland, such as articles by authors of books and pamphlets in my sample published in the general press and women’s magazines; documentaries on family planning produced under SCM supervision by the Educational Film Studio (Wytwórnia Filmów Oświatowych, henceforth EFS) in Łódź^50^ and a number of reports developed or published by SCM/SFP/SRR, the IPPF and the World Health Organisation.

## ‘Conscious Family Planning for Women’s Health’: Legitimising the Use of Contraception

In this section, I examine strategies deployed in popular medical literature to legitimise contraception as both a private activity and part of medical practice. Framing family planning as a tool to preserve the health of mothers, their families (defined as married couples with children) and society as a whole—an approach already used across the globe by 20th-century family planners attempting to mainstream their cause—was the most prominent of these strategies.^51^ Many SCM/SFP/SFD doctors, inspired by the Polish interwar tradition of birth control^52^ and their contemporary links to the IPPF, used this strategy to smooth the transmission of new, necessarily explicit information about sex and contraception: now part of population and public health policy. This transmission was aimed at a public heavily influenced by Catholic tradition, and SCM activists were fully aware of the Polish Church’s opposition towards ‘artificial’ birth control.^53^

In 1958, SCM produced the first edition of *How to Prevent Pregnancy* by the female gynaecologist, Jadwiga Beaupre, one of the founding and most active members of the Society, who had a leading role in the SCM family planning clinic in Cracow. In this pamphlet, one of the most widely circulated SCM publications, Beaupre declared that the Society’s intention was ‘to fight abortions and help society create as many families as possible who have as many children as they desire and are capable of raising, and no abortions on their conscience’.^54^ This idea, encapsulating one of the main arguments consistently employed by SCM to popularise contraception use, could be identified in the text of the 1956 abortion law itself. Like the Soviet abortion law enacted the previous year, Polish legislation paradoxically framed termination explicitly as a defence of family but implicitly as defence of motherhood. Legalising abortion and taking it from the ‘back-alley’ to the hospital would protect women from botched procedures that threatened their lives and future reproductive health and, by extension, the well-being of their families and society as a whole.^55^ At the same time, legal, medicalised abortion was by no means encouraged but rather consistently represented as a dangerous, harmful intervention, a ‘lesser evil’, preferably the last resort to be taken by women who had the desired number of children and were facing extreme poverty.^56^

An association of abortion with infertility, especially in reference to women terminating their first pregnancies and becoming unable to conceive later, was widely discussed in almost all literature popularising contraception in Poland between the mid-1950s and mid-1980s. Framed as a tragedy for both individual women, stripped from the joys of motherhood and often referred to as ‘cripples’,^57^ and the state deprived of future citizens,^58^ this association—which was also an association between (productive) femininity and motherhood—is by no means specific to state-socialist Poland and has taken a central role in anti-abortion discourse in various contexts. The possibility of abortion-induced infertility was exploited in films used by Margaret Sanger—one of the most famous proponents of family planning in the USA and globally—to popularise contraception use in the USA during the 1910s and in similar campaigns by Planned Parenthood in the early 1950s.^59^ Yuliya Hilevych and Chizu Sato have shown that post-abortion infertility turning women into ‘cripples’ was also the main propagandistic resource in the Soviet Union during the second half of the 1950s and the 1960s. However, Soviet popular medical discourses, as exemplified by Hilevych and Sato’s analysis of the health popularisation magazine *Zdorovie* during the 1960s, differentiated between ‘healthy’ and ‘unhealthy’ contraceptive methods. The pill, for instance, was considered the latter and its use was persistently discouraged.^60^ Polish popular medical literature, in contrast, consistently framed birth control as a means of protecting not only women’s fertility from abortion-induced sterility but also their general health (from too frequent births) and the health and well-being of their children (from being born into disease or poverty), without such clear-cut attribution of pathogenic properties to certain contraceptive methods.

The prescriptive literature on contraception attached birth control to the idea of a family rigidly defined as a married couple with a purposefully chosen number of children, neither too few, nor too many: a total of three was generally considered ideal. In its early years, the SCM insisted its publications and activities were directed at married people, perhaps in an effort to allay anticipated opposition from the Catholic Church. *Before and After Getting Married* (1959), one of the first books published by PZWL under the SCM brand, explicitly underlined the fact the Society did not provide advice about birth control to single people, ‘unless there were medical indications to do so’.^61^ While other books published in the late 1950s and 1960s were not as explicit about their advice being unsuitable for unmarried readers, those overtly aimed at a young (female) readership—such as Jadwiga Beaupre’s *Before You Get Married* (1963)—focused heavily on preparation for marriage and motherhood as the ‘natural’ site of sexual activity. This prioritisation of the married couple, exemplified by books such as *ABC of Married Life* (1969), by male gynaecologist Dr Zbigniew Sternadel, endured until the late 1970s, when contraception advice directed at single people began to circulate more widely. This shift was not without controversy, as demonstrated by the female gynaecologist Michalina Wisłocka’s sex manual, *The Art of Love* (1978).^62^ Anthropologist Agnieszka Kościańska has pointed out that the book, a nationwide bestseller in the late 1970s, only reached publication two years after the manuscript had been submitted in 1976, exemplifying the extent to which unmarried sexual activity continued to raise concern.^63^ While Wisłocka’s explicit advice on how women could enhance their sexual pleasure was contentious, the real controversy lay in her argument that premarital (but monogamous) sex was an important test of compatibility. Perhaps in anticipation of negative reactions to this claim, the book cover was adorned with a newly-wed couple.^64^ Indeed, much of the literature published during the late 1950s and 1960s venerated marriage as a fundamental part of human life, as well as a way of dignifying sexual urges,^65^ an idea that resonated with Catholic tradition and was explicitly developed in literature on marriage and parenthood developed by Catholic authors, such as Karol Wojtyła’s *Love and Responsibility*, aimed at religiously obedient Poles.^66^ While premarital sex continued to provoke discussion, the idea that the main aim of marriage—whether Catholic or civil—was procreation went unquestioned and as historian Natalia Jarska has shown, continued to be the core assumption in public discourse about marriage during Polish state socialism.^67^

The assumption that the main aim of marriage was procreation, however, needed reconciling with the seemingly conflicting notion of limiting births. In contraception advice literature, these ideas were accommodated through an ongoing reference to birth control as a tool for achieving well-being for individual families and society as a whole. This individual and social well-being was to be achieved by discouraging the less healthy or, more importantly, less affluent, from uncontrolled procreation while encouraging married couples with economic stability to have more children. These arguments echoed what had been understood in the interwar era as positive eugenics:^68^ indeed, the word ‘eugenics’ itself was not only used when referring to avoiding or limiting the reproduction of unhealthy individuals in some of the manuals discussed here but also in legal documents and Catholic family planning materials well into the 1960s.^69^

In popular literature on family planning, expectations about who should have children shifted alongside changes in population policy. Birth control was particularly recommended for impoverished women during the late 1950s and 1960s, in the belief they were producing the next generation of ‘miserable’ welfare-dependent adults, a ‘burden to society’ and ‘unable to look after themselves’.^70^ However, unlike state-socialist Hungary or Czechoslovakia, where the idea of the ‘poor’ whose procreation required taming became strongly racialised and incarnated in reproductive policies targeting the Roma population, considered backward and excessively fertile,^71^ Polish family planning literature was not explicit in linking the ‘poor’ with ethnicity or with urban or rural locations. Those Poles who were ‘young, healthy, and fit’ were encouraged to procreate throughout the entire period analysed in this article,^72^ and during the 1970s, in line with the new pronatalist population policy, advice literature explicitly targeted those who postponed or limited their reproductive capacities to protect their level of personal consumerism.

The tension involved in attaining the optimal number of children during the late 1950s is best exemplified in the popular books and pamphlets by the prominent (male) professor of gynaecology in Warsaw and founding member of the SCM, Jan Lesiński. In *Before and After Getting Married*, published in 1959, Lesiński argued that a child born into a poor family every year was a burden to the state, but, as he phrased it, for the ‘fittest’, procreation was a social duty:

There is no reason for women who are young, healthy, physically and psychically fit to use birth control. Preventing births for personal commodity or other egoistic reasons is not right. Young married couples should remember that children are the purpose of marriage and a social asset.^73^

Such eugenical notions, as historian Sylwia Kuźma-Markowska has shown, were also present in Lesiński’s manual on birth control aimed at doctors and medical students,^74^ published the same year and advertised in medical journals throughout the 1960s.^75^ It is worth underlining, however, that people’s freedom to make choices about their families and the role of doctors in helping to make these decisions informed ones were important facets of Lesiński’s reasoning.^76^ As long as individual choices intersected with the greater socialist good, ‘conscious motherhood’ policies were to be based not on coercion but people’s free will, which family planning advice literature could aspire to influence.

The tension between individual and societal family planning goals continued in advice literature during the 1970s. Warnings against using contraception for ‘egoistic’ reasons then became encased in a broader discourse surrounding decelerating population growth, a common topic in the general press in the first half of the decade that concerned communist and Church authorities alike.^77^ In a 1974 book with the unambiguous title *To Prevent or Terminate Pregnancy*, Barbara Trębicka-Kwiatkowska, a renowned professor of gynaecology and obstetrics based in Lublin and one of few women to become the head of a university clinic in state-socialist Poland,^78^ praised ‘conscious family planning motivated by concerns about women’s health and the proper conditions to bring up children’. However, she also condemned those who were ‘hostile towards having the number of children appropriate to their [high] living standard’.^79^ The problem at this time was deemed to be couples who rejected social and socialist expectations in favour of shallow consumerism. Although, as mentioned earlier, the two-child family had become an increasing trend in Poland during the second half of the 20th century, the ideal family, consistently portrayed in the literature analysed here, had three children. No advice was given, however, on how to reconcile a family of this size with paid employment or household chores.

## ‘May He See His Wife Always Pretty, Smiling and Smelling Fresh’: Femininity, Family Harmony and Physical Attractiveness

In the framing of contraception as a ‘modern’ or potentially modernising tool within the context of the present and future well-being of the family and the society, potentially more controversial arguments for family planning—such as women using contraception to attain self-fulfilment in their sexual lives and beyond—were rarely noted. In Britain, as Hera Cook has shown, the non-repression of sexuality was one of the key innovations for fertility control within marriage during the ‘long sexual revolution’ (1800–1975).^80^ Polish state-socialist advice literature only sporadically mentioned contraception as a way of improving a woman’s sex life, as well as that of the couple, and this was usually framed in terms of the negative impact that fear of an undesired pregnancy could have on both partners,^81^ and, in turn, on the family’s harmonious cohabitation.^82^ In pamphlets on birth control aimed at both professionals (1959)^83^ and the lay public (1965),^84^ Michalina Wisłocka claimed contraception prevented irritability and family tension. Wisłocka, involved with SCM since its foundation, had worked at the Society’s family planning clinic in Warsaw for several years, gaining the sexological expertise she deployed in *The Art of Love* (1978). In *Contraceptive Methods* published over a decade earlier (1965), Wisłocka had elaborated on the tensions that could result from a lack of contraception:

Continuous fear of pregnancy causes a woman to despise having intercourse with her husband, and the permanent nervousness and irritability of parents takes its toll on the family atmosphere.^85^

In a pamphlet for doctors, published in 1959, Wisłocka had painted a vivid picture of how employing birth control methods, particularly diaphragms and spermicides, could alleviate this situation:

… we could observe psychological changes a woman underwent once she started to control her fertility. Women who came [to the clinic] psychologically exhausted, in panic, broken, after a few months of following our indications become calm and serene, and the atmosphere of joy and harmony returned to their families.^86^

Contraception’s association with sexual pleasure was, for Wisłocka, directly linked to harmonious cohabitation within the couple and the family, a connection—as Agnieszka Kościańska has argued—the gynaecologist also utilised in her later works, including *The Art of Love*.^87^

Both male and female authors occasionally argued that contraception would enable women to attain autonomous, personal well-being and professional growth. Arguments regarding the latter were, however, marginal, appearing consistently only in pamphlets by Barbara Trębicka-Kwiatkowska—all published in the first half of the 1970s—who encouraged contraception use as a way for women to pursue professional careers^88^ or for her and her husband to complete a university degree.^89^

A more established argument associated a woman’s well-being with the ability to preserve her youth and beauty, thereby keeping her husband sexually interested and the marriage content. In 1961, for instance, female gynaecologist Joanna Tołwińska emphasised rational family planning as a means of avoiding hardship and preserving physical attractiveness by describing the tragic fate of a newly pregnant mother of seven:

This poor mother …. About to face sleepless nights, diapers to wash and permanent concern about what to put in her children’s mouth. Where is her time for rest, for entertainment? How can she buy a new dress, coat, shoes? And when she looks into the mirror, she will be unpleasantly surprised to see that her beauty, her light movements, her lovely smile which used to make her so popular—are all gone.^90^

This focus on a woman’s need to retain physical attractiveness—her key asset—was also a feature of advice literature by gynaecologist Jadwiga Beaupre. *How to Prevent Pregnancy* (1958), contained a chapter on ‘Women’s Hygiene’, with a lengthy discussion on the importance of women making the effort to look as ‘aesthetically’ pleasing as possible:

A woman, no matter how difficult her circumstances, should look after her appearance. May she look in the mirror at least once a day and try to look her best. … And as you should not show him anger, don’t show him dirty underwear, blood stains in the sheets. … May he see his wife always pretty, smiling and smelling fresh.^91^

In the subsequent editions of the book, in which several chapters, most notably the one on contraceptive methods, were substantially updated, the section ‘The Aesthetics of Woman’s Appearance’ remained unchanged.

According to a number of manuals, a woman’s physical attractiveness was not only spoiled by too frequent births and too many children but also the pregnancy itself. Beaupre believed a woman usually became ‘very ugly when expecting’^92^ and should make a particular effort to conceal her expanding and unalluring belly. This argument was remade two decades later, in 1978, in the aforementioned *The Art of Love*. In Wisłocka’s best-seller, pregnancy and the birth of the first child were presented as major threats to marriage, with young mothers concentrating all their efforts and attention on the new-born, neglecting their appearance and embarrassing their husbands.^93^ In such circumstances, husbands with high sex drives would certainly look elsewhere.

## ‘The Egoistic and Unreasonable Attitude of Men’: The Division of Responsibilities and Choice of Contraceptive Methods

The idea that women—and by extension their families—were to benefit from family planning was stable across the period analysed here and closely linked to the division of responsibilities in this realm. In this section, I examine how men were encouraged to take a more active role in family planning and how the gendered division of contraceptive labour impacted on the representations of different contraceptive methods.

The majority of books and booklets published in the late 1950s and during the 1960s and 1970s were explicitly aimed at women, an approach that endured in some of the most recent books in my sample: ‘When we talk about contraception we mean first and foremost women’, wrote the female gynaecologist Jadwiga Żywuszko in 1984.^94^ Women were consistently represented as the intended recipients of contraceptive advice; the Society would recommend a combination of diaphragm or cervical cap with a spermicide, especially during the late 1950s and 1960s. From the late 1960s onwards, the emphasis on female barrier methods gradually faded in favour of ‘modern’ contraceptives for women: the pill and, as a first choice, the intrauterine device.^95^

This focus on women echoes the traditional approach dating back to 19th-century British and American birth control advocates, such as Robert Dale Owen, George Drysdale and Charles Knowlton. Advice about contraception was targeted at women, who could then act independently of their husbands’ will to cooperate.^96^ As it was women who were faced with pregnancy, birth and childcare, Margaret Sanger believed they should also be responsible for contraception and possess effective means to prevent undesired pregnancies.^97^ The idea that it was women who should be educated about birth control also intersected and overlapped with a wider tradition of women being the recipients of health advice in general, due to their responsibility for healthcare and medication within the household.^98^

While it was taken for granted that family planning was a woman’s responsibility, contraceptive advice literature consistently encouraged men to take an active part in preventing unwanted pregnancies. Addressing men in birth control campaigns was by no means specific to state-socialist Poland. As historian Amy E. Randall has shown, the Soviet anti-abortion campaign of the mid-1950s and 1960s targeted male comrades, framing abortion prevention as a ‘husbandly concern and a family matter’.^99^ The campaign, Randall argues, was built upon a transformed model of Soviet masculinity with a newly recognised engagement and authority in a realm previously considered a woman’s domain. Yuliya Hilevych and Chizu Sato have demonstrated how, in the same period, the popular Soviet health magazine *Zdorovie* encouraged men to ‘protect’ women from unwanted pregnancy and abortion by also using condoms.^100^

A number of Polish doctors condemned the fact that contraception was viewed as ‘no man’s land’. In the expanded introduction to the third and fourth editions of *How to Prevent Pregnancy*, Jadwiga Beaupre described the fact that sex meant pleasure for men while women had to deal with any tough consequences by themselves as ‘a great social injustice’.^101^ In the only book to be explicitly aimed at men that I could locate, *Men’s Sexual ABC* (1963, 1965), written in a similar language to her publications aimed at women, Beaupre claimed greater male involvement in family planning would compensate for women’s almost sole responsibility for bearing and educating children and described the ideal 1960s man:

… a good husband, who loves his wife and children and has a sense of responsibility for their fate. This type of person will never turn away from his wife with an indifferent: ‘it’s your business, so handle it’. On the contrary, he will join his wife in facing the difficulties of this ‘downside’ to sexual cohabitation, which is regulating the number of children in a family.^102^

The tasks an ideal husband should undertake, according to Beaupre, included calculating fertile and infertile days, using condoms, which she considered the simplest and most secure methods, and, if contraception happened to fail, dealing with the paperwork required for an abortion. For Beaupre, ‘true masculinity in a world where women were able to study and support themselves’—a nod towards the state-socialist policy of sex equality—lay not in the husband providing financial support but in being responsible for his own actions, especially in the realm of sexuality.^103^

Opinions about men’s involvement in family planning not only appeared in *Men’s Sexual ABC*. Other medical authors of both genders contributed to the debate, which continued throughout the 1970s. The female gynaecologist, Janina Krocin-Karasek, author of a brochure for domestically manufactured intrauterine devices (IUDs) and contraceptive pills,^104^ acted as an expert on contraception in a cycle of 1974 articles published in *Przyjaciółka*, Poland’s most popular women’s magazine. In her emotional plea for men to take responsibility, Krocin-Karasek equated contraception with a man’s love for his wife:

Men and women are equally responsible for the fate of the family and for how numerous it will be. Those men who are deeply emotionally attached to their partners will want to protect them from termination and its harmful consequences.^105^

Some professionals linked this active role with the necessity of male cooperation in a number of recommended techniques, such as the rhythm method, and prioritised condoms as the most accessible and secure contraceptive.^106^ Other doctors took male involvement a step further and promoted ‘natural regulation of conceptions’, based on cycle-observation and abstinence, as an expression of spousal love, which, rather than avoiding or spacing births, involved co-responsibility for conscious conception. The aforementioned Włodzimierz Fijałkowski, along with authors of literature explicitly intended for Catholic spouses during the 1960s and 1970s, insisted that fertility awareness was a lifestyle of co-responsibility and mutual love, not a contraceptive method.^107^ Interestingly, somewhat similar arguments in favour of periodic abstinence as a natural, safe and responsible contraceptive method, especially for men, also appeared in Soviet popular medical discourses on birth control in the 1960s.^108^

Although Polish advice literature often encouraged men to take an active role in family planning duties, male contraceptive methods, especially the method most widely practised by Polish men during the 20th century, *coitus interruptus*, were criticised and pathologised. The constant pathologisation of withdrawal in the literature I examined was often paired with—and is symptomatic of—the persistent popularity of this contraceptive method in Poland, as documented in sociological surveys on contraceptive practices carried out in the 1970s.^109^ Withdrawal was also a prevalent method in other countries in the region, including Yugoslavia, Czechoslovakia, Bulgaria and the Soviet Union,^110^ as well as the sexual cultures of Catholic nations and communities throughout the world.^111^ In some contexts, such as early 20th-century Britain and communist Yugoslavia, scholars have interpreted the capacity to accomplish withdrawal as a cultural component of successful, caring masculinity. Yet, in Britain, the method continued to be condemned by the British birth control movement as being ineffective and highly likely to diminish women’s sexual pleasure.^112^

Polish advice literature almost always denounced withdrawal as a cause of serious health problems in both spouses, an argument present in West European prescriptive literature on contraception since the beginning of the 20th century,^113^ as well as the Soviet Union during the 1960s, where, as Hilevych and Sato have argued, it was linked to a threat as great to the gender script for masculinity as infertility was for women: impotence.^114^ With sporadic references to extremely serious ailments, such as heart attack or stroke, the main criticism attached to *coitus interruptus* in Polish popular medical texts was its association with unresolved sexual tension in both partners. In men, this tension resulted from the obligation of maintaining constant control and thus not benefiting from the relaxation sex could provide. An understanding of the sex act as something that is and should be uncontrollable was common in Polish sexological discourse during the 1970s.^115^ For women, the tension resulted from the prematurely finished act depriving them of orgasm. This, according to the authors of most contraception advice literature, led to physical symptoms such as pelvic congestion and psychological ones, namely a bad temper and irritability, potentially threatening family contentment.^116^ The exception was Wisłocka, who condemned withdrawal in her 1965 *Contraceptive Methods* but in the *Art of Love* (1978), claimed it was an innocuous contraceptive technique for ‘calm men’ in stable relationships (261–62). Advice literature did not cite empirical studies to support claims of a negative impact on men’s health from withdrawal: it appears that traditional ideas about withdrawal being unhealthy were perpetuated without scientific reflection.

The contraceptive that most divided opinion was the condom, which some authors presented as the best choice, while many others completely rejected the method. Jadwiga Beaupre’s *What Agatka Learned about Conscious Motherhood* (1961), for instance, a short story intended for rural women, about women receiving birth control advice in their village, featured a doctor who exclaimed ‘A woman whose husband uses condoms is a happy one’.^117^ A contemporary manual by (male) professor of gynaecology Tadeusz Bulski, however, presented condoms as unreliable, a problem raised in a number of books during the 1970s and 1980s, whose authors warned of the poor quality of domestically manufactured rubber.^118^ Aside from quality issues, doctors also claimed condoms diminished sexual pleasure for both partners,^119^ were aesthetically unpleasing^120^ and deprived women of the beneficial effect of semen on their bodies.^121^ A condom’s role in preventing venereal disease was almost totally absent from advice literature, and if mentioned at all, appeared in the context of ‘single men, who have love affairs and fear contracting venereal disease’.^122^

## ‘When a Man’s Drunk, He Doesn’t Think about Contraception’: Masculinity, Violence and Alcoholism

Not only were male-controlled contraceptive methods viewed with ambivalence, the ideal discussed earlier of a caring husband, actively involved in family planning, clashed violently with examples of husbandly behaviour put forward in popular literature on contraception. The key feature of this behaviour was egoism, which, as Natalia Jarska has suggested, typically appeared as a distinctive male flaw in broadly considered literature on marriage and relationships produced in state-socialist Poland.^123^ In regard to family planning, it manifested itself as indifference or even outright hostility towards contraception. As male gynaecologist Rafał Pumpiański wrote, in one of the first large print booklets on contraception (1957), ‘Regrettably, it is not unusual to see an extremely egoistic and unreasonable attitude in men who think that they are not obliged to take any precautions, because women have to manage “these things” on their own’.^124^ Nearly two decades later, these words were repeated almost literally in Barbara Trębicka-Kwiatkowska’s *To Prevent Pregnancy or to Terminate It*,^125^ illustrating the stability of this gendered metaphor of an egoistic husband evading his birth control responsibilities. This stability synchronises with Barbara Klich-Kluczewska’s description of Polish society under state socialism as static and unrevolutionary, with social change arrested by omnipresent poverty and developments taking place not over decades, but across half a century.^126^ Some authors linked such attitudes with a Catholic or traditional upbringing,^127^ while others condemned ‘egoistic’ men forcing legal abortion upon their wives as a low-cost solution.^128^

A far more frequent form of violence mentioned in the advice literature was the euphemistic ‘brutality’ of husbands: marital rape. In the medical birth control propaganda for a general readership that I analysed, the spectre of husbands ‘not respecting their wives’ and being ‘violent’ (the usual adjective is ‘gwałtowny‘, etymologically linked to ‘gwałt’ [rape]) is a permanent fixture throughout the period.^129^ One explanation for this ‘brutality’ appears in Dr Tadeusz Bulski’s *Guide to Marriage*. This book, which, as mentioned earlier, appeared in seven editions and almost half a million copies, depicts a series of consultations between a doctor and a 24-year old woman with three small children. During her first visit, the patient, who had undergone four abortions in rapid succession, seemed almost resigned to her fate of ‘many more pregnancies’:

Doctor, you know my husband, how violent and incomprehensive he is. He often drinks and then he would not care about anything. … I tried to talk to him but he says it’s my fault. … Doctor, you know it, when a man’s drunk, he doesn’t think about contraception. I tried to pretend I’m sick so we don’t [have intercourse] but he threatened he’d leave me.^130^

The situation described by Dr Bulski’s fictional patient illustrates dimensions of gender hierarchy and gendered sexual violence. First, the fact a male partner threatening to leave was enough for a woman to endure unwanted intercourse exemplifies an asymmetrical power relationship. Secondly, the doctor already ‘knew’ about the husband’s violent and ‘unsympathetic’ behaviour; indeed, the issue was raised in relation to family planning rather than a concern in itself, an example of the normalisation of abuse highlighted in Barbara Klich-Kluczewska’s work on the (non)-tabooisation of ‘domestic violence’ in state-socialist Poland.^131^ This normalisation, as anthropologist Agnieszka Kościańska has also shown, was linked with the naturalisation of male sexuality as primitive, savage and uncontrollable, an ongoing idea in sexological discourse during the last two decades of state socialism.^132^

The narration by Dr Bulski’s patient also brings out another element of this uncontrollable male sexuality: the consumption of alcohol, often considered the main catalyst of violence and another factor normalising men’s irresponsible behaviour. Alcoholism was an immense and increasing public health and social problem in state-socialist Poland^133^ and, as Barbara Klich-Kluczewska has argued, became a convenient explanation for domestic violence during the 1970s, ghettoising the phenomenon and preventing victims from receiving real support from the state and perpetrators from being punished.^134^ Likewise, alcohol overconsumption was inextricably linked to a man rejecting birth control or ‘forcing intercourse’, being considered to both provoke and practically justify his actions. This link remained stable in books and pamphlets on contraception published throughout the period analysed here, from the mid-1950s to the mid-1980s.^135^ Given Dr Bulski’s failure to address abuse in his fictional consultations, it appears the naturalisation of alcohol-incited sexual violence by husbands had also infiltrated medical practice. Certainly Bulski, and most of his male and female colleagues who wrote about contraception for a general readership at that time, merely recommended the patient use a contraceptive method that did not require male cooperation.

During SCM’s early years, this option was a combination of diaphragm or cervical cap and spermicide. In the journalists Maria Karaś and Hanna Polsakiewicz’s advice booklet *Letters to a Friend* (1961, 1962), published by SCM, a woman encourages her friend to conduct her contraception measures clandestinely:

You can use contraception that he won’t notice. You have to remember to wear the diaphragm. He won’t feel it. I don’t see the need to talk about this with my husband. You may not talk about it to yours either, perhaps sometime later.^136^

Towards the end of the 1960s, medical literature popularising birth control switched allegiance from diaphragms to the IUD as the contraceptive method of self-defensive preference.^137^ In an article on Polish IUDs published in *Przyjaciółka* in 1976, Dr Mieczysław Cisło gave his reasons for frequently prescribing the coil:

I cannot prescribe oral contraceptive pills to women after many abortions, whose husbands don’t cooperate, force intercourse or are alcoholics. These women work hard, they work multiple shifts, they have several children and husbands who only think about themselves. I cannot be sure these women would be taking pills regularly, will remember to take them every day. I offer them Spider CU [a Polish IUD].^138^

Diaphragms and IUDs created a barrier, if not from rape, then at least against any resulting offspring or abortions, and along with high efficacy, had the additional advantage of not requiring daily compliance.

## Conclusion

The contraception advice literature published in Poland between the legalisation of abortion in 1956 and the mid-1980s exhibits the gendered tensions, contradictions and hierarchies involved in constructing family planning as a legitimate medical and social activity. The arguments used for this construction were driven by ideas about population management on both sides of the Iron Curtain. Polish authors strategically employed arguments relating to the health and prosperity of the entire family, tactically attempting to subdue social anxieties relating to public intervention in birth control practices, previously the almost exclusive domain of the Catholic Church.

While rejecting coercion—from the state or individuals, such as husbands encouraging their wives to terminate pregnancies—advisors consistently criticised affluent, ‘egoistic’ spouses, especially wives, for not producing enough children, while encouraging poor women to refrain from bringing offspring into the world that would burden the state. One may wonder how this advice applied to Polish society, where living standards were generally low throughout the period analysed. Only in the 1970s did the Polish United Worker’s Party place more emphasis on consumerism and access to consumer goods. At the same time as this change was taking place, family planning literature produced by Catholic doctors was elevated to ‘official’ status and mainstreamed through the PZWL.

Advice literature timidly and belatedly—from the 1970s onwards—began to place less emphasis on family planning as something pertaining exclusively to married couples. Concurrently, family planning advice materials began to advocate contraception for reasons that could be perceived as controversial or ‘egoistic’, such as a woman’s professional and sexual fulfilment (usually framed in relation to the happiness of the couple or family as a whole). Throughout the entire period under analysis here, family planning was generally represented less as a tool enhancing gender equality in terms of women’s professional roles and more in terms of men’s responsibilities towards their wives and children.

Similarly to the representation of gender roles in popular sexological literature during the 1970s and 1980s, publications on contraception for a general readership took legal equality between the sexes for granted yet failed to apply this to gender relationships as performed through the practices of sex, reproduction and contraception. Gynaecologists who wrote about family planning for the general public from the 1950s through to the 1980s tended to portray the relationship between the sexes as complementary, with most attaching responsibility for contraception to those who bore the consequences of unwanted pregnancies: women. These included abortion, unequivocally represented as dangerous and potentially sterilising, to the almost exclusive (and unquestioned) obligation to care for resulting children. At the same time, advisors put pressure on men to become more involved in family planning practices, while simultaneously discrediting and pathologising the traditional male birth control technique, *coitus interruptus*, and constructing an ambiguous portrayal of condoms. While preaching co-responsibility for contraception, this literature also acknowledged the omnipresence of gender violence, condemning ‘silly, egoistic’ husbands, incited by alcohol into forcing sexual intercourse on their spouses and recommended that women could and should use contraception without their husband’s knowledge. While framing contraception as strategically useful, these advisors bestowed new knowledge upon women on how to manage birth control, sanitised and legitimised this knowledge and, in their own way, encouraged women to react against established and largely unquestioned gender hierarchies by rejecting, if not undesired intercourse, then the possible consequences.

A direction for future research that emerges from this study is the establishing of a dialogue between ‘official’ discourses on contraception as represented in popular medical literature and those developed in Catholic marital literature and marriage preparation materials. The circulation of these narratives increased considerably from the late 1960s onwards, a time when reducing the number of abortions through the mainstreaming of ‘natural regulation of conceptions’ became a key task for the Polish Episcopate. Another profitable area for research would be the actual impact of these discourses on Polish birth control practices: to that end, I am currently developing an oral history project.

## Funding

This research was funded by the National Science Centre (Poland) Polonez grant (ref. 2016/21/P/HS3/04080). This project received funding from the European Union Horizon 2020 research and innovation programme under the Marie Sklodowska Curie grant agreement no. 665778. The Gold Open Access fee was covered by a Microgrant from the University of Warsaw Rector (awarded on 25 March 2019).