# Corrigendum to: No Man's Land? Gendering Contraception in Family Planning Advice Literature in State-Socialist Poland (1950s–1980s)

**DOI:** 10.1093/shm/hkz114

**Published:** 2019-12-06

**Authors:** Agata Ignaciuk

*Social History of Medicine*. doi:10.1093/shm/hkz007

In the original published article, reference 36 has been changed to read: Piotr Perkowski, ‘Wedded to Welfare? Working Mothers and the Welfare State in Communist Poland’, *Slavic Review*, 2017, 76, 455–80, 471. This error has now been corrected.

